# Supplementary material for: Continuous odor profile monitoring to study olfactory navigation in small animals
Source: eLife. 2023 Jul 25;12:e85910. doi: 10.7554/eLife.85910 (PMC10425172; doi:10.7554/eLife.85910)
Supplement: Supplementary file 1. — Also available at https://doi.org/10.6084/m9.figshare.21737303. [file elife-85910-supp1.pdf]

Software Setup

Software requirements

Arduino IDE 1.8.16 or later, with Teensyduino 1.55 or later

LabVIEW 2021 or later 32-bit, with NI-VISA and NI-Serial drivers

MATLAB R2019b recommended, but other recent MATLAB versions should work too.

Labjack driver for LabJack-U3. One can use NI-DAQ and replace Labjack if available.

Installation

Clone the following repository and its submodules to your local machine:

- Odor sensor array: https://github.com/GershowLab/OdorSensorArray
- Labjack LJUD: https://github.com/Kevin-Sean-Chen/LabVIEW_LJUD/tree/develop

Follow PJRC's guide to load the sensor control code **OdorSensorArray/arduino_data acquisition teensy code/osa/osa.ino** to Teensys, note down the COM port used by each Teensy. For Labjack control, connect U3 labjack and run **LabVIEW_LJUD/Examples/U3 /U3 Read Serial Number.vi** to identify the COM port. In the same folder, run **MFC_PID_flow_new.vi** to control and record from MFC and PID.

Hardware Setup

Ordering Parts

The odor sensor array PCBs are designed using AutoDesk EAGLE.

- OdorSensorBar (OSB) is printed and assembled by MacroFab. The design file **OdorSensorArray/eagle_PCB design files/OdorSensorBar.brd** is already formatted to be compliant with MacroFab's EAGLE guideline. Each OSB contains 16 odor sensors (OS) and 8 temperature and relative humidity sensors (HS in this repository; THS in the publication).
- OdorSensorHub (OSH) is printed by OSH Park. The design file **OdorSensorArray/eagle_PCB design files/OdorSensorHub.brd** is in compliance with OSH Park's EAGLE design rule files.

You can use other PCB printing and assembly services, but you might need to modify the design files to be compliant with their specific guidelines, and you might need to produce a separate Bill of Materials (BOM) for OSB.

Other electronic parts, including 2 Teensy 4.0 microcontrollers, are ordered separately; please refer to **OdorSensorArray/eagle_PCB design files/odor sensor array_parts list.xlsx** for details.

For airflow tubings, machined parts, mass flow controller (MFC) and photoionization detector (PID), please check the detailed parts list: http://doi.org/10.6084/m9.figshare.21737303

Manually soldered connections

The 4-pin right-angle headers (H0) should be soldered to the bottom layer of OSB and the corresponding 4-pin right-angle sockets (B0-6) should be soldered to the top layer of OSH, as shown below:

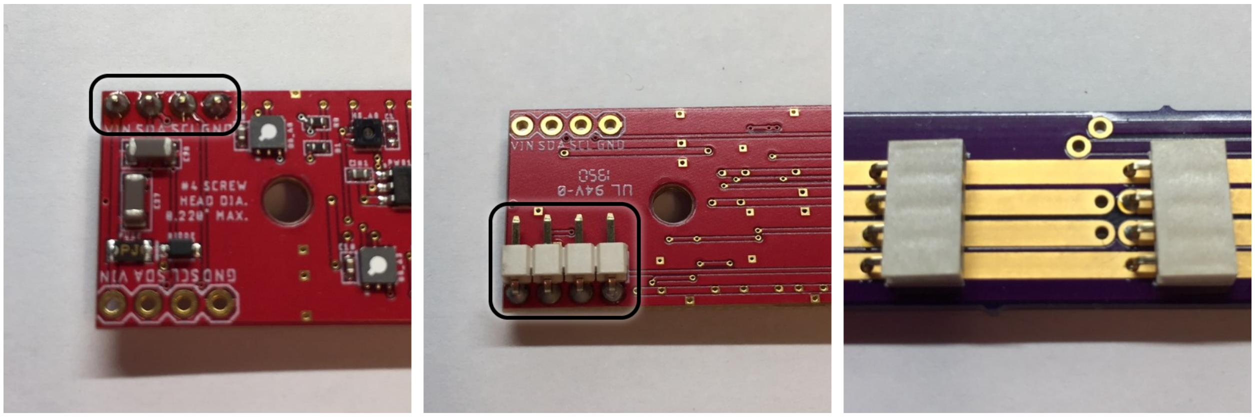

Up to 7 OSBs can be plugged into 1 OSH with these right-angle connectors. Alternatively, if you need more flexibility in sensor locations, you can use a 4-pin pogo pin header to connect an OSB to anywhere on the exposed copper rails on the OSH. See **Mounting OSBs** for details on securing the boards in place.

The data (SCL/SDA0-1, single cable with a 4-conductor core for easy wiring) and power cables (VDD, GND) connecting OSH and the breadboard should be soldered to the labeled vias on OSH. Since the vias are very close to each other on OSH, we recommend using heat shrink tubing to insulate them from each other, and using non-conductive tape to protect the solder joints on the bottom of the board from touching any conductive surface.

Breadboard connections

The Teensys should be connected to OSH as shown below; each Teensy should be connected to and powered by the computer via a micro USB cable, which is not shown here.

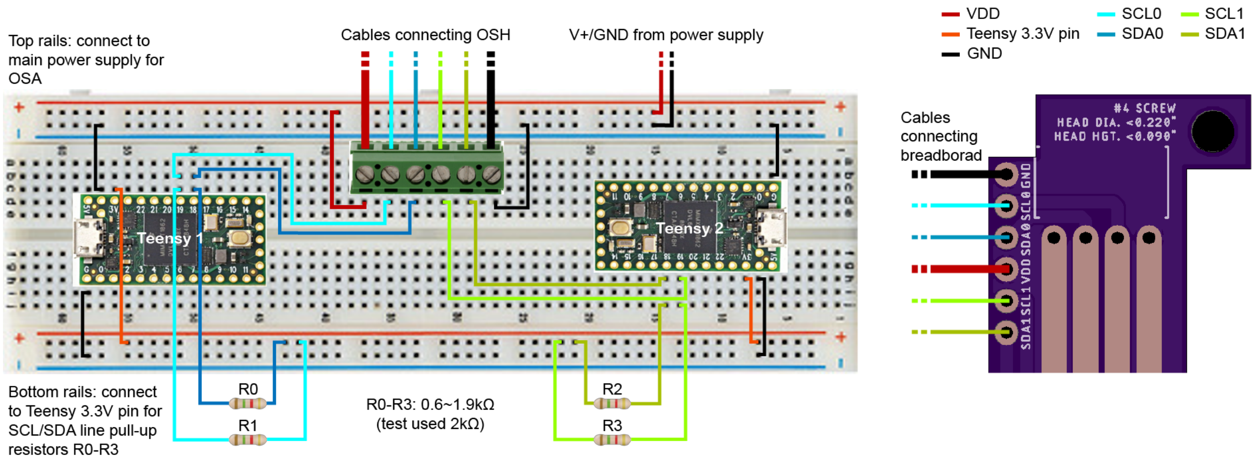

Mounting OSBs

Each odor sensor contains a micro hot-plate which heats up during measurements. To dissipate this heat, you should mount the OSBs onto a metal heat sink, and pre-treat the bottom side of the OSBs with thermal paste and heat transfer tape to help with heat dissipation.

Plug OSBs into OSH, set the 2-digit address for each OSB on the physical DIP switch located at one end of the board as shown below.

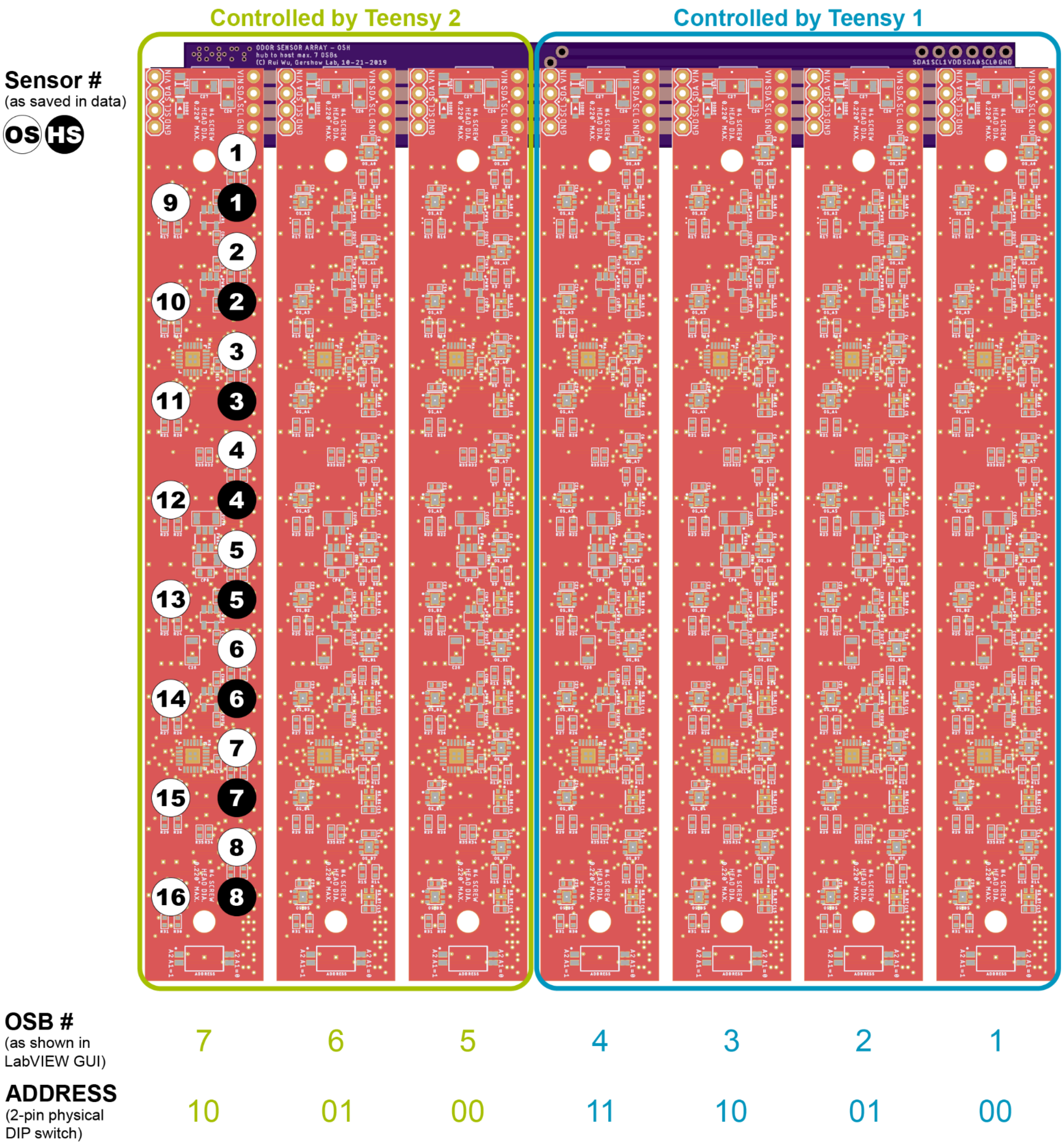

2-pin DIP switch setup example:
ADDRESS = 01 means pin A2 = 0, A1 = 1 as shown →
follow silk screen, ignore digits printed on the switch itself

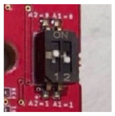

Secure each OSB with 2 #4 screws (max. screw head diameter 0.220”) to the heat sink below.

Providing power to OSA

For a single OSB, the typical working current and voltage for a single OSB is 0.7A at 2.2V; max. allowed supply voltage is 3.6V. Given the internal resistances of power cable and breadboard components as well as how many OSBs are plugged in, there will likely be an additional voltage drop ΔV from the power supply to the OSH once the sensors are powered up and drawing current.

We suggest using a variable DC power supply unit capable of supplying at least 3.5V/5A. Before powering up, make sure the total internal resistance between the power supply unit and the VDD/GND pins on OSH is less than 0.3Ω, so that maximum possible ΔV for the 7-OSB mode is not too large that you ever need to supply more than 3.6V and risk damaging the circuit. In practice, we've found that supplying 2.6V/2.3V works well enough for 7-OSB/2-OSB modes respectively.

Control MFC and measure with PID

After connecting MFC and PID to the Labjack, run the vi:
LabVIEW_LJUD/Examples/U3/MFC_PID_flow_new.v

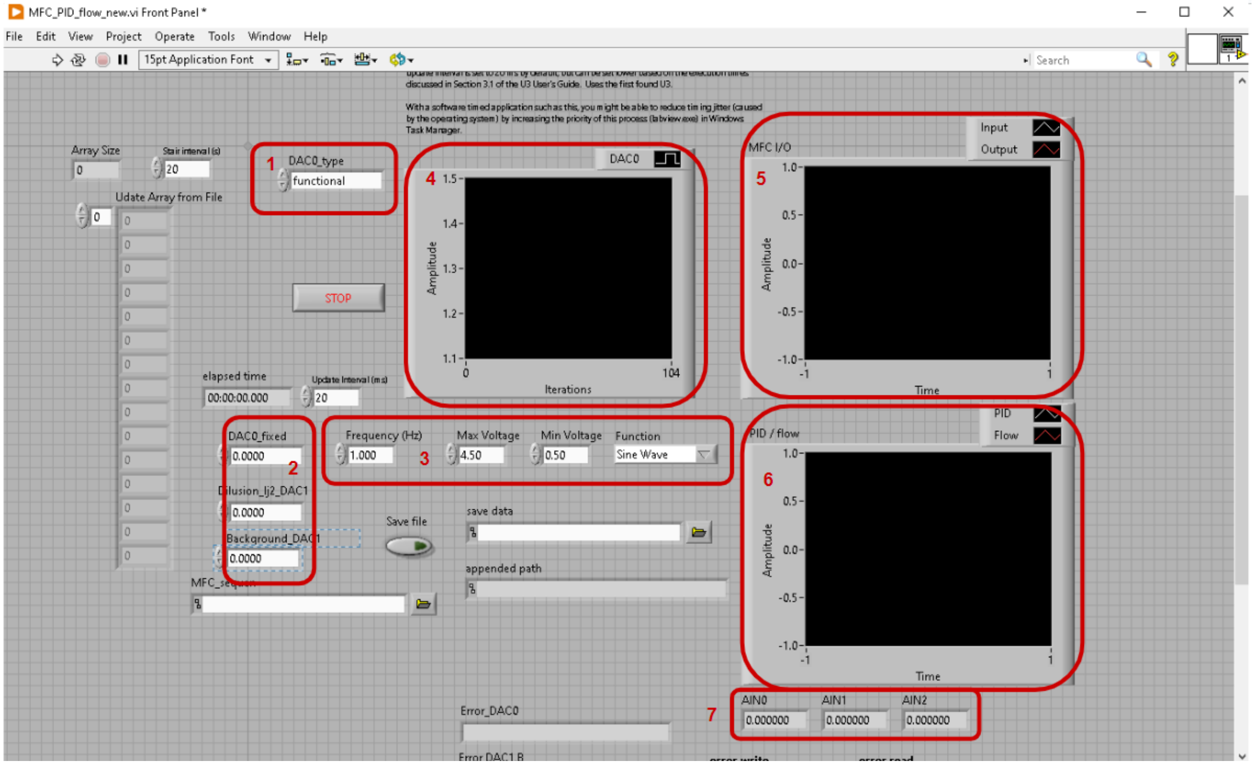

Controlling MFC

1. Select the type of MFC control protocol: functional, fixed, read. The ‘functional’ option is used to create waveforms from a functional generator, such as the triangle waves used for sensor calibration. The frequency, waveform, and amplitude are controlled by (3). For ‘fixed’ protocol, the voltage for three MFCs connected are shown in (2). For ‘read’, one can fill in the voltage in the array on the left. This is used for step-wize waveforms.
2. Voltage to the MFC connected to odor (DAC0), gas-phase dilution (this is optional), and background airflow (DAC1).
3. Used when (1) is selected as ‘functional’.
4. Real-time voltage of the controlled odorized airflow
5. Real-time traces of the controlled (input) and readout (output) voltage to the MFC.
6. Downstream PID voltage (PID) and the optical sensor from the flow meter (flow).
7. Raw readout from PID (AIN0), MFC control (AIN1), and MFC read (AIN2). These pin numbers can be changed dependending on the connection ports to LabJack.

Measuring PID

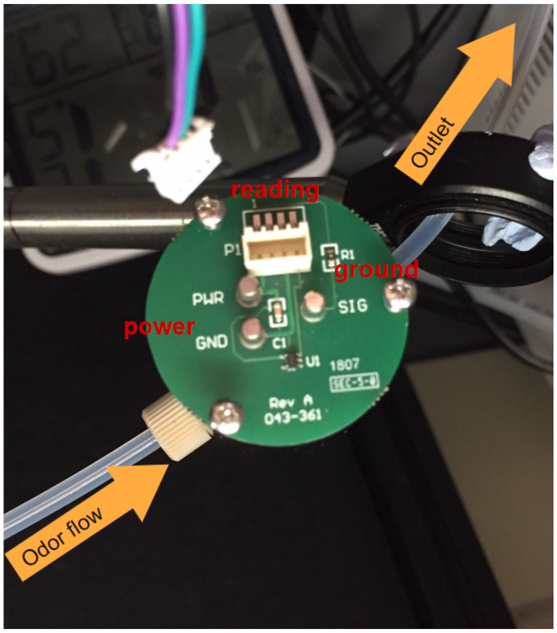

We connect PID with the piD-TECH piD-POD chamber. The output from the flow chamber is connected to the POD and output into the lab. We solder wires to power (5V) and read from the PID (connected to AIN0).
The data format saved by MFC_PID_flow_new.v has 6 columns:

1. Computer clock (1ms)
2. PID readout (AIN0)
3. MFC control (AIN1)
4. MFC readback (AIN2)
5. MFC for background airflow (DAC1)
6. Optic sensor from a downstream flow meter

Taking Odor Measurements

Using the LabVIEW data acquisition GUI

Once all connections are made and power is supplied to OSA, open **OdorSensorArray/labview_data acquisition GUI/osa_main.vi**:

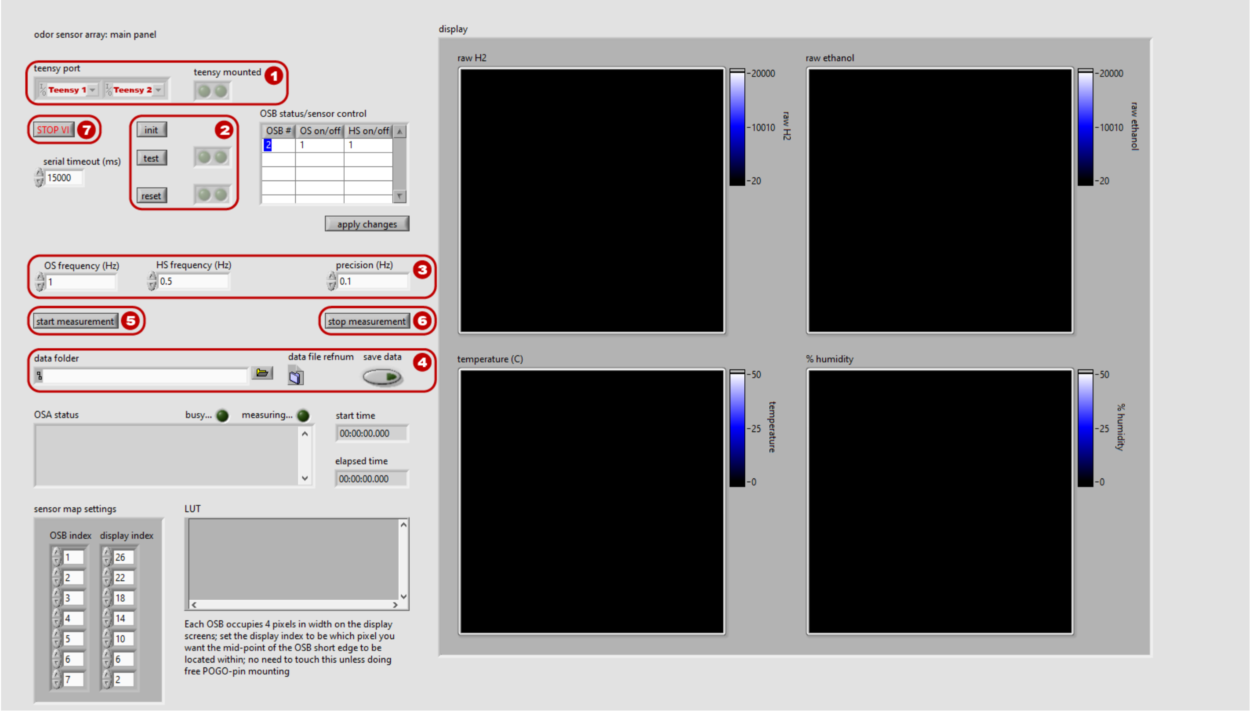

1. Select the corresponding COM ports for Teensy 1 and 2, then run the VI; make sure the “teensy mounted” indicator light is green for both Teensys once the VI is running.
2. Click “init”, “test”, and “reset” in this exact sequence, wait for indicator lights to go green for each step before proceeding to the next.
    - If you are monitoring voltage and current on the VDD pin on OSH, you should see that once “test” is clicked, current will increase in ~0.7A increments as each OSB is powered on and running the test sequence, and voltage will drop in steps accordingly, eventually stabilizing at ~2.2V. See [Providing Power to OSA](#) for details.
    - If idled for too long, sensors can drop into sleep mode and stop drawing current, and you may see voltage and current fluctuations on the VDD pin accordingly. This is ok.
3. Set desired sampling frequency for odor and humidity sensors, and adjust “precision (Hz)” to reflect precision of the smaller number - e.g. if OS frequency = 1Hz and HS frequency = 0.05Hz, then precision should be 0.01Hz.

- Attention: maximum frequency allowed is ~2Hz with all 7 OSBs plugged in and both OS and HS measurements are taken, and can be faster with fewer sensors with some modifications to the Arduino code.

4. If saving data to disk, select the folder to save the .txt file in, and click on the “save data” button to enable data saving.
5. Click “start measurement” to begin data acquisition.
6. Click “stop measurement” to end data acquisition. As long as the VI is still running, you can take multiple datasets by clicking the “start/stop measurement” buttons without repeating steps 1-4.
7. When done, click the “STOP VI” button to release the COM ports from LabVIEW’s control and terminate the VI.

Data format

Sensor data is saved to disk in a tab-delimited text file with 10 columns:

1. ms_timer: LabVIEW clock (ms) used for syncing with other types of data
2. teensy_timer: Teensy clock (ms) used for syncing and latency checks
3. code: 0 = start measurement, 1 = OS readings, 2 = HS readings
4. bus_num: 1 = Teensy 1, 2 = Teensy 2
5. osb_index: 1-7 matching the OSB # shown in **Mounting OSBs**
6. sensor_index: 1-16 for OS, 1-8 for HS; matching the OS/HS # shown in **Mounting OSBs**
7. sensor_ID: unique serial ID for each sensor for troubleshooting
8. data_1:
    1. if code = 1 (OS readings): 16-bit H2 raw reading
    2. if code = 2 (HS readings): temperature (°C)
9. data_2:
    1. if code = 1: 16-bit EtOH raw reading
    2. if code = 2: relative humidity (%)
10. valid: 0 = readings are invalid, 1 = valid; used for troubleshooting

See **OdorSensorArray/matlab_data analysis/demo/simple_odor_flow.m** for a demo on reading and processing sensor data in MATLAB.

Sensor Calibration, Stationary Flow, and Pre-equilibration Protocol

Sensor calibration protocol

The metal-oxide sensors are exposed to the same odorized air flow as the photo-ionization detector (PID). The readout is calibrated by aligning output from both detectors. During calibration, we use all inlet and outlet ports with tubings connected to the same manifold. This ensures that the effective resistance is the same and we can control the spatially uniform flow reliably. The time varying air flow is controlled by a mass flow controller (MFC) bubbling a bottle with diluted odorant. Another MFC provides background moisturized air at a higher flow rate. These two paths are connected with T-shape connectors before passing to the manifold. In our setup, the background moisturized air is at 400 ml/min and the time varying flow rate normally ranges between 0-50 ml/min. This configuration allows us to control odor concentration through air flow but not significantly changing the overall flow rate (~10% variation). Since metal oxide sensors are at different positions in the arena and PID is downstream from the full sensor array, there is a time delay between these measurements. The program computes the delay time and computes the mapping between metal oxide sensors and PID readings offline.

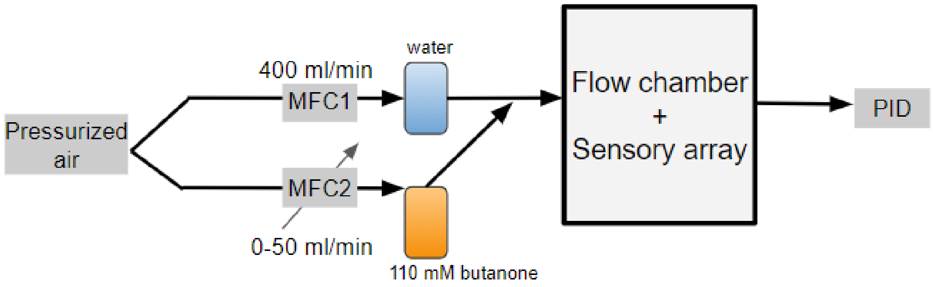

Schematic for tubing connection for sensor calibration

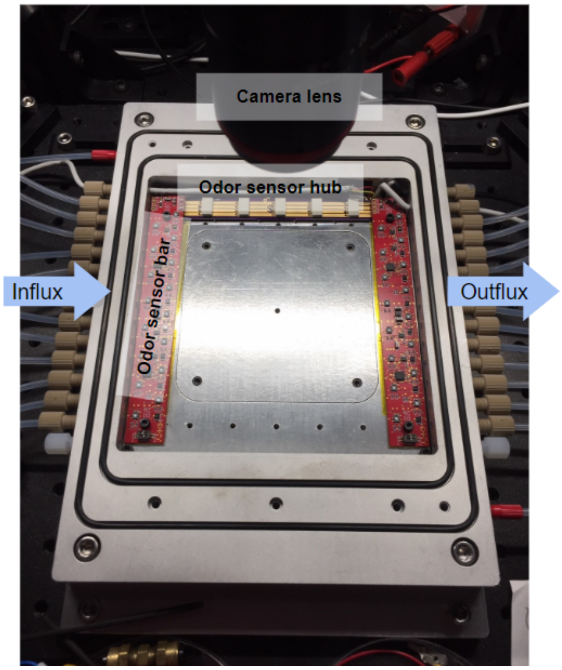

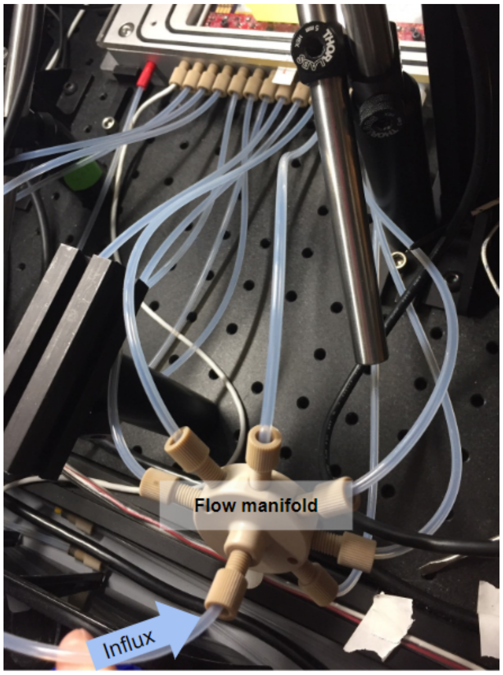

Left: top view of the chamber with tubing connections. Right: flow manifold to distribute flow.

The goal for this calibration protocol is to (1) map the odor sensor readout to ppm values from PID, (2) ensure that the prepared target odorant can reliably produce odor in air, and (3) prove

that there is no additional residuals, hysteresis effect, or air leaking in the system. We measure stationary odor landscape and conduct animal experiments after the calibration.

Step-by-step guide:

1. Connect all tubings in and out of the flow chamber

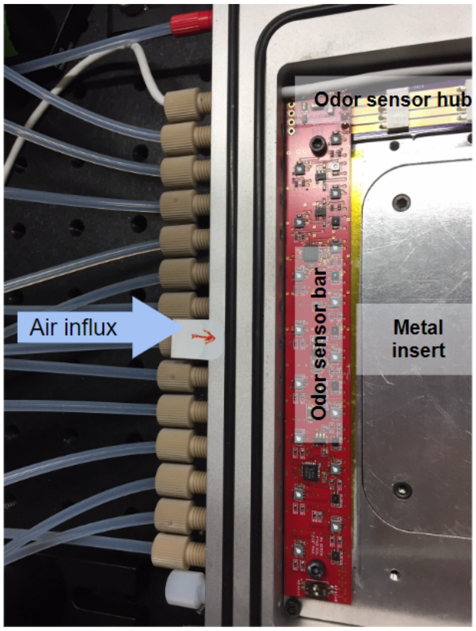

2. Prepare the odor bottle with the target odorant and another bottle with water for moisturize background air and connect them to the flow path

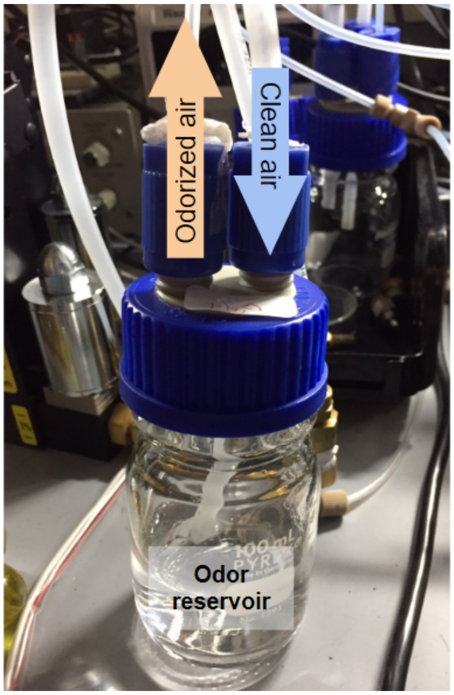

3. Turn on air flow and vacuum seal the flow chamber
4. Make sure that the water and odor bottles are bubbled and that the downstream flow meter has the correct readout, showing that there is no leak.

5. Turn on and initialize the odor sensor array (also see Taking Odor Measurements):
    a. Load Arduino code **OdorSensorArray/arduino_data acquisition teensy code/osa/osa.ino** to both Teensy. Make sure that the booting light flashes then it is done loading.
    b. Turn on the power supply to the OSA board.
    c. Start Labview code **OdorSensorArray/labview_data acquisition GUI/osa_main.vi**. Select the two Teensy connections and run.
    d. Initialize the sensors, test, and then reset the sensors. Make sure that the sensors are drawing current from the power supply and are stable.
    e. Start recording from the OSA
6. Start recording the odor sensors for 5 minutes of baseline values
7. Start the time-varying uniform odor flow through MFC control. We use 0.002 Hz (500 second period) triangular waves altering the odorant flow rate. Make sure that the maximum concentration readout does not exceed the maximum reading for PID.
8. Save both the OSA and PID tables and run **OdorSensorArray/matlab_data analysis/scripts/OSA_PID_map.m** script to compute the calibration curve for each sensor
9. These calibration curve are stored as a structure and later on used to convert sensor array readings to ppm values

Controlling steady-state odor landscape

Configure the inlet tubings to form different odor landscapes:

- Centered-cone shape: connect the odorant tubing to the middle port and the remaining tubings with moisturized air to the sides
- biased cone shape: bias the odorant tubing to the side
- Inverse cone shape: swap the odorant inlet and the clean air tubings
- Altering the gradient along cone shapes: one can change the relative flow rate between the odor source and background air flow to adjust the “width” of these cone shapes, thereby changing the landscape gradient.

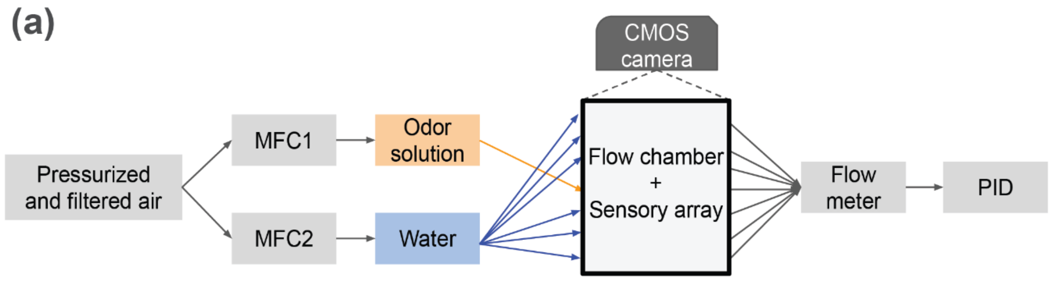

Figure 1a shown in the main text. This connection is used to construct odor landscapes.

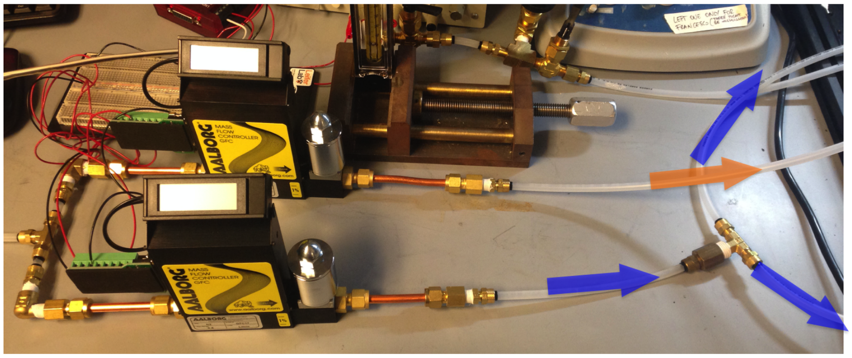

Two mass flow controllers: the upper one controls flow into the odor reservoir (orange arrow). The lower one carries moisturized background air (blue arrows)

After configuring the inlets, we turn on both MFCs connected to clear air and to the bottle of odorant. In our experiments, we normally use 400 ml/min moisturized air flow as the background and ~33 ml/min for the odorant bottle. Without agar in the chamber, in real time one can monitor the downstream PID readout increasing and reaching steady-state value in 5-10 minutes, as shown in the supplementary figures.

Pre-equilibration protocol and animal experiments

The pre-equilibration protocol is used for odorants that have significant interaction with agar, such as butanone that is highly soluble in water. Without this protocol, the downstream readout is distorted when we introduce a piece of agar in the arena for animal experiments. We account for this deviation from steady-state by bringing the agar plate close to the equilibrium pattern quickly. This protocol starts with the same flow parameters as what we use to form steady-state odor landscapes, but with higher concentration in the odorant bottle. In practice, our target odorant bottle with butanone is 11 mM in water and the high-concentration bottle we apply is 110mM butanone in water.

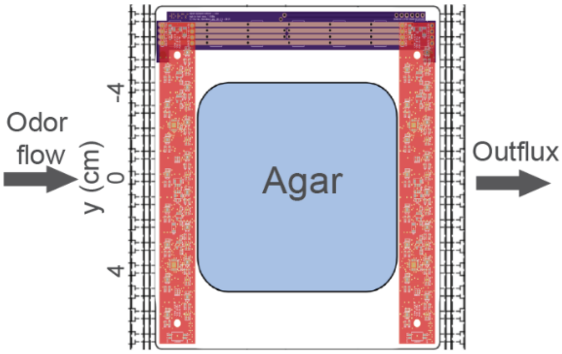

Configuration shown in figure 4 in the main text: the middle insert contains an agar plate and the two sensors are measuring boundary conditions.

With the same configuration as steady-state odor landscape experiments, the high-concentration odorant (110 mM butanone) increases concentration measured by the downstream PID. When this readout surpasses the steady-state concentration measured

without agar, we swap the odorant bottle back to the target concentration (11 mM butanone). This can be done with a toggle or directly changing the bubbling odor source. The experimental results show that the final concentration is close to stationary and presents less than 10% drift across the 30 minute recording of behavioral assays. When the boundary odor bars have the same readout as those shown without plate, this means that there is no additional absorption or emission from the plate during equilibrium. We conduct this protocol for each new agar plate before each animal experiment.

Step-by-step guide:

1. Remove the middle 5 odor sensor bars and the metal insert. Fixate the plate of agar in the insert and make sure that two boundary odor sensor bars are fixed on the surrounding insert.

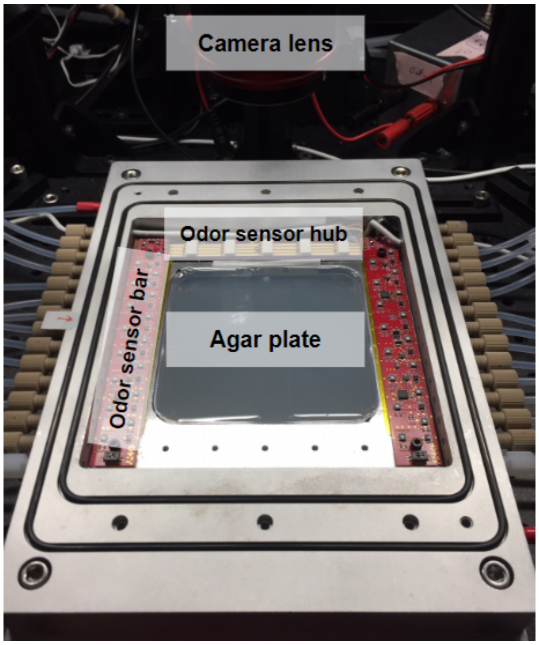

2. Connect tubings following the configuration used to form steady-state odor landscapes
3. Connect the high-concentration odorant bottle to the MFC for odor control and a bottle of water to the MFC for background flow
4. Initialize and start recording with the two boundary odor sensor bars
5. Start odor flow from the high-concentration odorant bottle and observe the readout from downstream PID in real time
6. When the downstream PID reports concentration that is approximately or slightly larger than steady-state values without agar plate (around 50 ppm for 11 mM butanone), disconnect the tubing going into the high-concentration bottle and reconnect it to the working concentration bottle
7. The downstream PID value would fluctuate for a few minutes due to the reconnection procedure, but after 5-10 minutes, it should stabilize around the target concentration, indicating that the agar plate has reached quasi-equilibrium with the odorant in air in the flow chamber

8. Animal experiments can be carried out after the quasi-equilibrium is reached. For larvae, we directly place animals down to the plate without removing it. For worms that are smaller and require adjustment under the microscope, we take out the plate temporarily and drop worms when we observe under the scope. In either case, when the plate is fixated and the acrylic lid is vacuum sealed again, the system takes ~3-5 min to come back to steady-state with our working concentration
9. Proceed to turn on the infera-red LED for dark-field illumination and start the behavioral imaging camera after the lid is sealed. Focus images on the agar plate surface and tune the LED intensity to adjust contrast of the worm body.

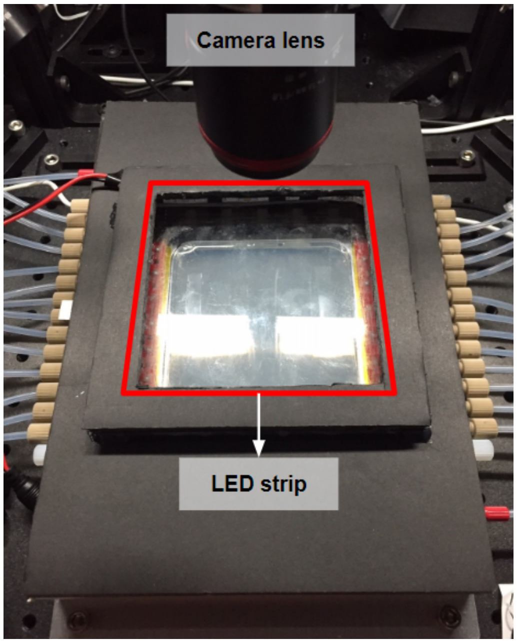

Cleaning and maintenance

In all experiments, from calibration to animal experiments with flow, one should always turn off the odorant airflow and continue with moisturized clean air afterwards. We observe the downstream PID readout and wait until it relaxes back to baseline (~0.1V) value. This is crucial to prevent any residual molecules in the chamber from causing contamination and also checks if the molecule might have interaction with the flow chamber or tubings.
After ensuring that the chamber is clean, we turn off the pressurized air source and vacuum source. Turn off electronics (control and reading for MFC, PID, and the sensor array). Disconnect the odorant and water bottle and wipe off the solution on the bubbler tubings. If liquid/condensation from either water or the odorant bottle enters the flow path, terminate the air flow and disconnect all tubings. Apply strong airflow to clean out droplets along the flow path.

References and Notes

Sources of AutoDesk EAGLE part libraries used in the designs (add the folder **OdorSensorArray/eagle_PCB design files/libraries/** to EAGLE's search path for easier access):

- MF_Passives.lbr is downloaded from MacroFab EDALibraries
- SparkFun-*.lbr are downloaded from SparkFun Electronics Eagle Libraries
- Parts in SamacSys_parts.lbr are acquired using the electronic component search engine provided by SamacSys
- Parts in Rui-Parts.lbr are created by Rui Wu

The manufacturer of the humidity sensor ENS210 has changed from ams to ScioSense after the circuit boards were made. The GitHub repository **OdorSensorArray** includes the original ams datasheet used in the design process, but the manufacturer part number and technical details have not changed.
